# Which factor affects the storage of real-world object information in visual working memory: perceptual or conceptual information?

**DOI:** 10.3389/fpsyg.2023.1239485

**Published:** 2023-10-18

**Authors:** Qiankai Li, Zhen Chen, Qi Sun, Xinyu Li

**Affiliations:** ^1^College of Psychology, Zhejiang Normal University, Jinhua, China; ^2^Key Laboratory of Intelligent Education Technology and Application of Zhejiang Province, Zhejiang Normal University, Jinhua, China

**Keywords:** visual working memory capacity, perceptual information, conceptual information, categorization, real-world objects

## Abstract

Visual working memory (VWM) is a limited dynamic memory system where people temporarily store and process visual information. Previous research showed that real-world objects do not have a fixed capacity compared to simple ones. In konkle’s study, they found that the conceptual information and perception information of real-world objects had different effects on visual long-term memory (VLTM) capacity. VLTM capacity was more dependent on conceptual information than the perceptual distinctiveness of real-world objects. However, we did not know how the intrinsic attribute of real-world objects affects VWM capacity yet. In the current research, we set five experiments to explore the comparative effects of conceptual vs. perceptual information of real-world objects in VWM capacity. Our results suggested that VWM capacity was more dependent on the perceptual distinctiveness of real-world objects than on conceptual structure. These data provide evidence that VWM capacity for real-world objects depends more on perceptual information than on conceptual structure.

## 1. Introduction

Visual working memory (VWM) is an active, limited memory system that processes visual information, and stores it for a short interval (Baddeley and Hitch, 1974; Cowan, 2008). Although there has been abundant research on visual working memory exploring the storage mechanism of simple objects such as colored squares or polygons (e.g., Luck and Vogel, 1997; Vogel et al., 2001; Wheeler and Treisman, 2002; Zhang and Luck, 2008), these studies have shown that VWM is a capacity-limited memory system.

In contrast to the simple, meaningless objects in the laboratory environment, the real-world objects people encounter in daily life are considered to be rich in semantic information (e.g., Brady et al., 2008). Brady et al. (2016) first used real-world objects as experimental stimuli to explore differences between simple and real-world objects in the storage mechanism of VWM. Their results showed that in an experimental condition of prolonged encoding time, the storage capacity of real-world objects in visual working memory was significantly better than that of simple ones. Brady et al. (2016) argued that these results suggested that visual working memory was not fixed. Perhaps conceptual information related to real-world objects or the involvement of the VLTM system led to these results. Li et al. (2020) used the same paradigm to replicate Brady et al. (2016) research. However, the results showed that simple and real-world objects improved VWM capacity as the encoding time increased. Consistent with Li et al. (2020) results, Quirk et al. (2020) failed to replicate the behavioral and contralateral delayed activity (CDA) results of Brady et al. (2016). This inconsistency suggests that the VWM storage mechanism of real-world objects should be further explored.

Natural objects contain more rich conceptual and perceptual information than meaningless simple ones. Konkle et al. (2010) used real-world objects of different categories to explore the long-term memory storage mechanism. They found that the conceptual structure of real-world objects represented by categorical information supports detailed visual long-term memory much better than perceptual structure does. Konkle et al. (2010) defined conceptual information and perceptual information as the categorical information (i.e., subordinate category structure for one given object) and shape or color information of real-world objects, respectively. In their research, the categorical information was ranked to distinctiveness or similarity according to the number of the kinds of a given object category (e.g., the number of kinds of cars) from 200 object categories in which each category contained 16 exemplars. For example, participants should rate how few or many kinds of cars and bowties there were, and based on the rating results they thought there were many kinds of cars but few kinds of bowties. That is, cars belonged to the conceptual distinctiveness condition and bowties belonged to the conceptual similarity condition in Konkle et al. (2010) research. They found that the objects belonging to the conceptual distinctiveness condition showed less interference in VLTM as the number of exemplars increased than the conceptual similarity condition. Brady et al. (2011) argued this result could result from conceptual hook effects which means the category information existing in VLTM could provide a cue to enable the recovery of a memory trace to benefit the memory performance. These effects improve performance in the storage and retrieval of objects containing rich conceptual information. Similarly, Koutstaal et al. (2003) found that the memory performance of real-world objects which were conceptually rich was better than that of perceptually rich objects. Though these results might suggest that the subordinate category information could be used to support episodic long-term memory (LTM) better than perceptual information for real-world objects, some questions remain unanswered. *How do the conceptual and perceptual structures of real-world objects play their roles in visual working memory? Do participants utilize conceptual information about real-world objects stored in LTM to support VWM storage?*

In the current study, we addressed this problem through five experiments by change detection task (CDT) deemed as a classical paradigm to explore the VWM capacity (e.g., Phillips, 1974; Luck and Vogel, 1997; Brady et al., 2016). In Experiments 1 to 3, we explored the effects of color and shape information about real-world objects on VWM storage. We subsequently investigated the effects of conceptual information about real-world objects on VWM storage in Experiments 4 and 5.

## 2. Experiment 1

In Experiment 1, we explored the effects of color information on real-world objects in VWM storage. We adopted a single factor design with two levels, *color-distinctive* and *color-similar* conditions.

### 2.1. Methods

#### 2.1.1. Participants

We recruited 25 participants (three males; mean age: 20.0 years) with normal and correct-to-normal vision and normal color discrimination abilities from Zhejiang Normal University. All participants were required to complete a practice testing before the formal testing. If the accuracy in practice testing were equal to or less than the chance level (i.e., 50%), the participants would be excluded from our research. The experiment was fully approved by the Scientific and Ethical Review Committee of the Department of Psychology of Zhejiang Normal University. The participants signed an informed consent form.

#### 2.1.2. Apparatus and stimuli

All the participants were required to place their chins on a chin rest during the testing phase. The distance between the participant’s eyes and the center of the screen was 60 cm. All stimuli were presented on a 17-in CRT (refresh rate: 60 Hz; resolution: 1600 × 900). The experiment was carried out in a dimly lit laboratory. Material presentation and response collection were executed via Python scripts.

**FIGURE 1:**
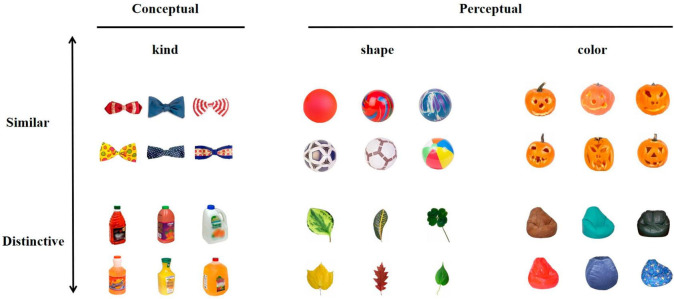
The example of distinctiveness rating. Images reproduced and adapted with permission from The Vision and Memory Lab (https://bradylab.ucsd.edu/stimuli.html).

**FIGURE 2:**
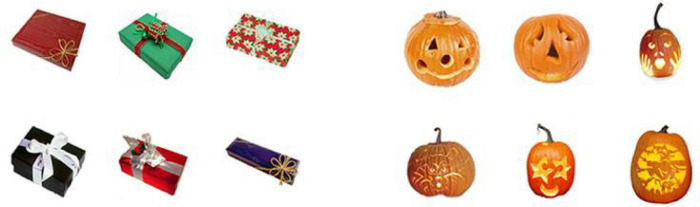
The partial exemplars of real-world objects used in the present experiment. The exemplars on the **left** belonged to the *color-distinctive* condition and on the **right** belonged to the *color-similar* condition. Images reproduced and adapted with permission from The Vision and Memory Lab (https://bradylab.ucsd.edu/stimuli.html).

We selected real-world objects from the stimuli pool of Konkle et al. (2010), downloaded from the web.^1^ In their study, a set of 200 object categories of real-world objects with 17 exemplars each was used for the task. Participants were instructed to rate the real-world object exemplars in each category on a scale from 1 (*very similar*) to 5 (*very distinctive*) for three distinctiveness dimensions (i.e., kind, shape, and color) by clicking a numbered button on the screen. For example, for color ratings, participants should rate how similar or varied the colors were between all exemplars from a given real-world object. As the sketch map shown in Figure 1 which was similar to Konkle et al. (2010), distinctiveness ratings were collected from all 200 real-world object categories and they acquired rating scores along each dimension (i.e., kind, shape, and color) as the basis to distinguish the distinctiveness. In the present experiment, we selected the objects with category ratings for a color dimension of fewer than 2 points as the optional categories for the *color-similar* condition. We selected those with a category rating for a color dimension of more than 4 points as the optional categories of *color-distinctive* conditions. There were two pairs of matched categories selected from the pool: buttons (kind dimension scores: 1.9; shape dimension scores: 2.6; color dimension scores: 5.0) matched with camcorders (kind dimension scores: 2.2; shape dimension scores: 2.7; color dimension scores: 1.6), gifts (kind dimension scores: 1.4; shape dimension scores: 2.4; color dimension scores: 4.5), matched with jack o’lanterns (kind dimension scores: 1.3; shape dimension scores: 2.7; color dimension scores: 1.0). Every object category had 17 exemplars. The Figure 2 illustrated the part of exemplars used in Experiment 1 and 2, and all the stimuli in the whole research could be downloaded (see text footnote 1).

#### 2.1.3. Procedure

**FIGURE 3:**
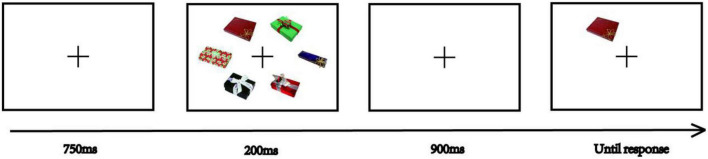
An example of a sequence of one trial event. Images reproduced and adapted with permission from The Vision and Memory Lab (https://bradylab.ucsd.edu/stimuli.html).

Figure 3 illustrates the procedure. The fixation cross was presented for 750 ms at the beginning of one trial. To exclude cross-category effects in VWM storage (Awh et al., 2007), that is, when sample and test objects belonged to different categories on change detection task the estimated VWM capacity was higher when sample and test objects belonged to same categories, in this memory stage, there would be six exemplars randomly selected from one object category. The exemplars were displayed on an invisible circle with a 3° radius visual angle even around the screen center for 200 ms. Each image was subtending a 2° × 2° visual angle. Next, there was a blank screen for 900 ms. In the test phase, the participants judged whether the probe object selected from the same category as the memory stage object was the same as the one presented at the same location in the memory stage by pressing the “Z (*no*)” and “/(*yes*)” keys on the keyboard. Participants were instructed that the reaction speed of reaction was not as important as accuracy. Participants were asked to press one key from the “Z (*no*)” and “/(*yes*)” keys compulsively even if they could not remember the sample. After the key was pressed, an intertrial interval would be lasting between 450 and 700 ms randomly. The next trial then began.

Each participant was asked to complete 12 trials in the practice testing and 64 trials divided evenly into 2 blocks in the formal testing. In each block, each category object used as the optional stimuli appeared at the same time (e.g., the number of occurrences of buttons and camcorders was evenly divided in each block). The trials on the response phase in the change and no change conditions occurred an equal number of times. All variables were randomly balanced across trials. The data from the practice phase were not included in the final results.

### 2.2. Results and discussion

**FIGURE 4:**
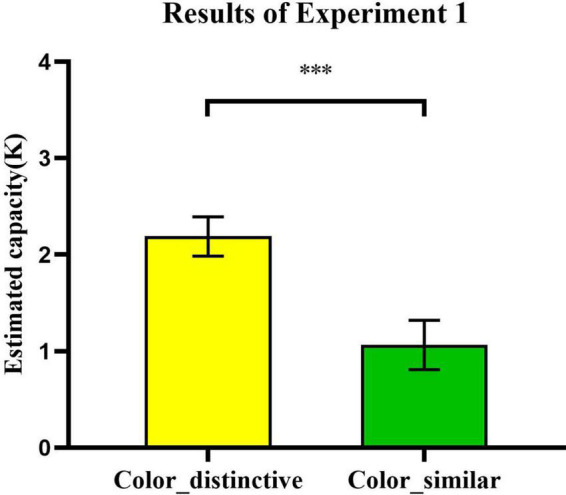
Estimated capacity (*K*) of VWM in *color-distinctive* and *color-similar* conditions. Error bars reflect standard error of mean. *, 0.01 < *p* < 0.05; **, 0.001 < *p* < 0.01; ***, *p* ≤ 0.001; and ****, *p* ≤ 0.0001.

We used Cowan (2001) as the VWM performance evaluation indicator for single-probe recognition which meant the estimated number of objects stored in VWM (Rouder et al., 2008; Rouder et al., 2011): *K*_*estimated*_ = *N* (*h*_*observed*_ − *f*_*observed*_) where *h*_*observed*_ and *f*_*observed*_ were observed hit and false alarm rates and *N*was the number of the to be encoding items (i.e., in the current research, the number of encoded foils was six). In addition, JASP (Version 0.17.1, JASP Team, 2023) were used to provide Bays factor to obtain a RELATIVE probability that the data provide evidence in favor of the alternative hypothesis as opposed to the null hypothesis (H1 vs. H0). BF_10_ stands for the Bayesian Factor. The subscript “10” means the likelihood that H1 can be true relative to that of H0. The larger the BF_10_ is, the more strongly the data favor the H1 (Wagenmakers et al., 2017). *K*-values were analyzed with paired-samples *t*-tests between *color-distinctive* (*Mean*: *K* = 2.19, *SEM* = 20%) and *color-similar* conditions (*Mean*: *K* = 1.07, *SEM* = 26%). *K*-values were significantly higher in the *color-distinctive* than *color-similar* conditions, *t*_(24)_ = 3.94, *p* = 0.001, Cohen’s *d* = 0.79 (Figure 4), BF_10_ = 56.316. These results suggested that color information affects VWM capacity. In addition, people maintain real-world objects containing distinctive colors much more easily than objects with similar ones. These results differed from those of Konkle et al. (2010). We thought that the observed difference in the two conditions could be potentially attributed to difficulty of distinguishing similar-colored objects from both the encoding phase and the decision-making phase. Eng et al. (2005) found that as the perceptual complexity of objects increased, the encoding limitations in the change detection task would be increased. Awh et al. (2007) found that more complex objects would entail high sample-test similarity so that performance would be limited during the comparison stage of the task. For the reason aforementioned, we suppose that the ability of participants in the encoding phase and decision-making phase is restricted when similar-colored objects were presented in the change detection task. Although the observed difference in the two conditions from Experiment 1 might be attributed to different VWM phases, these results could still prove that our visual working memory ability is impacted by color information in real-world objects more compared to VLTM. The perceptual dimension of the color of real-world objects did not correlate with the degree of interference in VLTM (Konkle et al., 2010). However, it did affect VWM storage in the current study.

## 3. Experiment 2

Brady et al. (2016) found the storage capacity of real-world objects increases compared to simple objects as encoding time increases. Although the present study did not focus on the effects of encoding time on VWM capacity, Brady et al. (2016) result inspired us to investigate if the representation of real-world objects in VWM storage might vary by encoding time. Therefore, in the present study, we further explored the stability of the results in Experiment 1 by setting different encoding times on the basis of Experiment 1. We referred to the previous research which used the change detection task to investigate the object on VWM storage and these studies have proved that 100 ms was adequate for encoding objects into VWM (Luck and Vogel, 1997; Vogel and Machizawa, 2004). Thus we set a short encoding time condition (100 ms) to further investigated the stability of the result from Experiment 1. To exclude the limitation of insufficient encoding and ensure the participant could observe the detailed information on real-world objects, we referred to the manipulation from Brady et al. (2016) and set a long encoding time condition (1000 ms).

### 3.1. Methods

#### 3.1.1. Participants

Twenty-five participants (six males; mean age: 19.92 years) participated in the current experiment. Participant requirements were identical to Experiment 1.

#### 3.1.2. Apparatus and stimuli

The apparatus and stimuli were identical to those used in Experiment 1.

#### 3.1.3. Procedure

**FIGURE 5:**
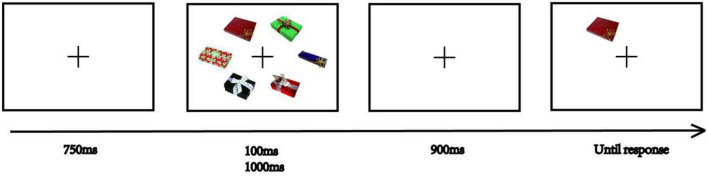
The Experiment 2 procedure. Images reproduced and adapted with permission from The Vision and Memory Lab (https://bradylab.ucsd.edu/stimuli.html).

The procedure was identical to Experiment 1 except with regard to encoding time and the number of trials. We set a shorter encoding time condition (100 ms) and a longer encoding time condition (1000 ms) than was used in Experiment 1 (Figure 5). All six exemplars on the sample display phase were presented simultaneously. Each participant first completed a practice section with 16 trials and then two blocks of 96 trials each in the formal experiment.

### 3.2. Results and discussion

**FIGURE 6:**
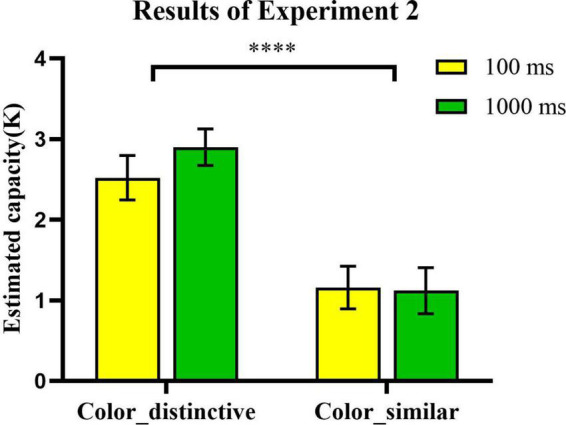
The effects of color distinctiveness and encoding time on the VWM task performance. Error bars reflect standard error of mean. *, 0.01 < *p* < 0.05; **, 0.001 < *p* < 0.01; ***, *p* ≤ 0.001; and ****, *p* ≤ 0.0001.

We conducted a 2 × 2 (encoding time × type of real-world objects) repeated measures analysis of variance (ANOVA) to examine whether the type of real-world objects in VWM storage differed by encoding time (Figure 6). Consistent with Experiment 1 results, the effect of type of real-world objects was significant [*F*_(1_, _24)_ = 52.00, *p* < 0.0001, η^2^_*p*_ = 0.68, BF_10_ > 1000]. The estimated *K*-values for the *color-distinctive* condition (*Mean*: *K* = 2.71, *SEM* = 19%) were significantly higher than the *color-similar* condition (*Mean*: *K* = 1.14, *SEM* = 23%). There were no significant differences across encoding times [*F*_(1_, _24)_ = 0.66, *p* = 0.425, η^2^_*p*_ = 0.027, BF_10_ = 0.332]. Our results were inconsistent with those of Brady et al. (2016), they found a significant estimated capacity increase with the encoding time increased from 200 to 2000 ms. We suggest that the disappearance of encoding time effects here resulted from floor effects in the *color-similar* condition. We suggest that objects belonging to the *color-similar* condition might contain more informational load and required more encoding time. The informational load from remembered objects (e.g., colored squares, polygons, cubes, etc.) was proved to account for about 99% of the variance in VWM capacity in Alvarez and Cavanagh (2004). Eng et al. (2005) further proved that the informational load was a better predictor of the estimated VWM capacity at a shorter display duration. Though the estimated VWM capacity for those complex objects (e.g., faces, cubes) could increase as the memory display duration increased, the estimated capacity of those complex objects were still lower than simple objects (e.g., colored squares) when display duration was 1000 ms (Eng et al., 2005). No significant interaction effects between encoding time and type of real-world objects were found [*F*_(1_, _24)_ = 0.76, *p* = 0.393, η^2^_*p*_ = 0.031, BF_10_ = 0.427]. These results demonstrate that the perceptual information about real-world objects’ colors affects VWM capacity.

## 4. Experiment 3

In this experiment, we explored whether the effect of perceptual information about real-world object shape on VWM storage was the same as that of color information.

### 4.1. Methods

#### 4.1.1. Participants

Twenty-five participants (three males; mean age: 18.68 years) took part in this experiment. The requirements (i.e., exclusion criteria for this experiment)for subjects were identical to Experiment 2.

#### 4.1.2. Apparatus and stimuli

**FIGURE 7:**
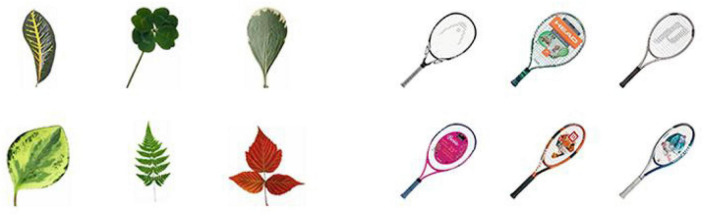
The partial exemplars of real-world objects used in Experiment 3. The exemplars on the **left** belonged to the *shape-distinctive* condition, and those on the **right** belonged to the *shape-similar* condition. Images reproduced and adapted with permission from The Vision and Memory Lab (https://bradylab.ucsd.edu/stimuli.html).

Apparatuses were identical to Experiment 2. A crucial difference was the stimuli. As Figure 7 shows, we selected two pairs of matched categories of real-world objects which contained distinct values in only the perceptual shape dimension as the stimuli in the current experiment and we presented the partial exemplars in Figure 7: trophies (kind dimension scores: 3.2; shape dimension scores: 4.6; color dimension scores: 3.2) matched with cigarettes (kind dimension scores: 3.2; shape dimension scores: 1.4; color dimension scores: 3.0), leaves (kind dimension scores: 2.8; shape dimension scores: 5.0; color dimension scores: 3.3) matched with the tennis racquet (kind dimension scores: 2.7; shape dimension scores: 1.6; color dimension scores: 3.8).

#### 4.1.3. Procedure

The current procedure was identical to Experiment 2.

### 4.2. Results and discussion

**FIGURE 8:**
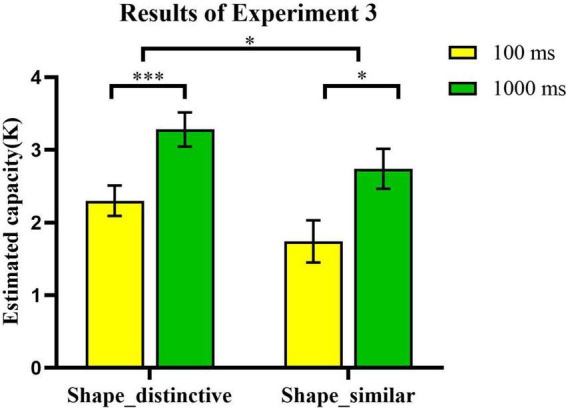
The effect of shape distinctiveness and encoding time on the VWM task. Error bars reflect standard error of mean. *, 0.01 < *p* < 0.05; **, 0.001 < *p* < 0.01; ***, *p* ≤ 0.001; and ****, *p* ≤ 0.0001.

We explored in a straightforward fashion shape information’s effect on VWM storage for different encoding times. As Figure 8 shows, a 2 × 2 (encoding time × type of real-world objects) repeated measures analysis of variance (ANOVA) was constructed. We found a significant main effect of the perception information of shape [*F*_(1_, _24)_ = 7.18, *p* < 0.013, η^2^_*p*_ = 0.23, BF_10_ = 2.669]. The memory performance in the shape-distinctive condition (*Mean*: *K* = 2.79, *SEM* = 20%) was better than the shape-similar condition (*Mean*: *K* = 2.24, *SEM* = 21%). We also found a significant main effect for encoding time [*F*_(1_, _24)_ = 18.49, *p* < 0.001, η^2^_*p*_ = 0.44, BF_10_ = 93.940]. The capacity of VWM was higher when the encoding time was 1000 ms (*Mean*: *K* = 3.01, *SEM* = 22%) than 100 ms (*Mean*: *K* = 2.02, *SEM* = 20%). Those results showed that shape information of real-world objects affected our VWM capacity. Capacity increased as the encoding time increased (Brady et al., 2016). There were no interaction effects between encoding time and type of real-world objects in this experiment [*F*_(1_, _24)_ = 0.002, *p* = 0.961, η^2^_*p*_ = 0.00, BF_10_ = 0.257].

## 5. Experiment 4

We found that the perceptual distinctiveness of real-world objects had a significant impact on VWM capacity, which was inconsistent with Konkle et al. (2010) research on VLTM. These results revealed that VWM and VLTM might have different storage mechanisms. In the current experiment, we explored the role of conceptual information about real-world objects in VWM storage. We adopted the operational definition of conceptual information of real-world objects used in the research of Konkle et al. (2010). Thus, we focused on the category distinctiveness of real-world objects on VWM capacity.

### 5.1. Methods

#### 5.1.1. Participants

Twenty-five participants (five males; mean age: 20.24 years) took part in the current experiment. The requirements for subjects were identical to Experiment 1.

#### 5.1.2. Apparatus and stimuli

**FIGURE 9:**
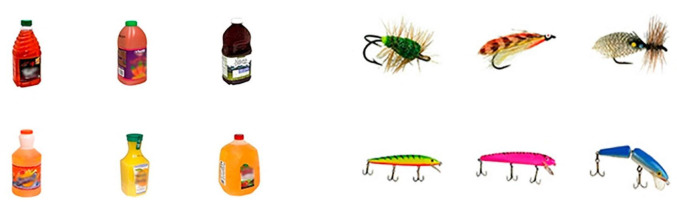
The partial exemplars of real-world objects used in Experiment 4. The exemplars on the left belonged to the *category-distinctive* condition, and on the right belonged to the *category-similar* condition. Images reproduced and adapted with permission from The Vision and Memory Lab (https://bradylab.ucsd.edu/stimuli.html).

The apparatus was identical to Experiment 1. We followed the operational definition from Konkle et al. (2010) and selected real-world objects according to conceptual distinctiveness rankings from Konkle et al. (2010). In Konkle et al. (2010) research, they specifically targeted the subordinate category structure to definite the concept of conceptual distinctiveness, that is, the number of kinds of a given object category (i.e., the variety of kinds). For example, there are many kinds of juice and only a few kinds of fishhooks just as Figure 9 shows. In Experiments 4 and 5, we controlled the *category-distinctive* condition and the *category-similar* condition by using the conceptual distinctiveness rankings results from Konkle et al. (2010). In the present experiment, we selected the objects with category ratings for the category dimension of fewer than 2 points as the alternative stimuli for the *category-similar* condition, and we selected those with a category rating for the category dimension of more than 4 points as the alternative stimuli of *category-distinctive* conditions. Finally, three matched pairs of real-world objects were selected in this procedure, bread loaves (kind dimension scores: 4.0; shape dimension scores: 3.5; color dimension scores: 1.9) matched with headphones (kind dimension scores: 1.6; shape dimension scores: 3.1; color dimension scores: 1.7), juice (kind dimension scores: 4.5; shape dimension scores: 4.0; color dimension scores: 4.6) matched with fishhooks (kind dimension scores: 1.6; shape dimension scores: 4.2; color dimension scores: 4.7), soda cans (kind dimension scores: 4.3; scores of the dimension of shape: 1.5; scores of the dimension of color: 4.9) matched with scrunchies (elasticized fabric rings used to style hair) (kind dimension scores: 1.1; shape dimension scores: 1.5; color dimension scores: 4.8).

#### 5.1.3. Procedure

The current procedure was identical to Experiment 1. However, we set 2 blocks of 72 trials each in the formal testing phase.

### 5.2. Results and discussion

**FIGURE 10:**
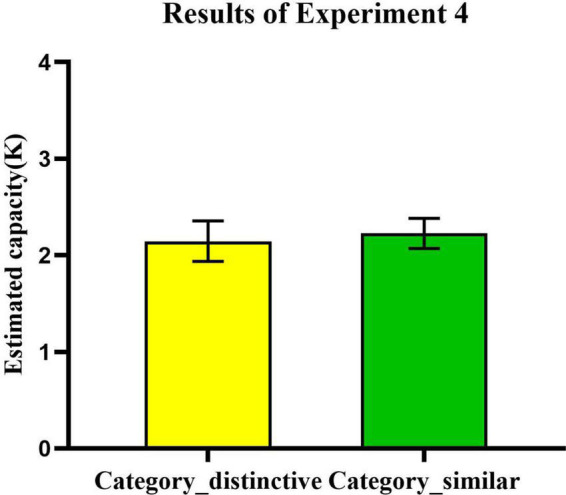
The effect of conceptual distinctiveness on the VWM task. Error bars reflect standard error of mean.

As Figure 10 showed that there was no significant main effect of conceptual information on memory of real-world objects [*t*_(24)_ = 0.37, *p* = 0.714, Cohen’s *d* = 0.07, BF_10_ = 0.225]. Our results concerning VWM were inconsistent with those of Konkle et al. (2010). The performance on real-world objects which were conceptually distinctive (*Mean*: *K* = 2.15, *SEM* = 21%) was not better than the objects which were conceptually similar (*Mean*: *K* = 2.23, *SEM* = 16%) in the VWM task. These results suggest that the VWM does not utilize passive information (i.e., category information) in VLTM. If category information could play a role as a cue on the VWM process to benefit the retrieval from LTM as Konkle et al. (2010) research shows, we should observe a significant VWM improvement from *category-distinctive* condition. However, our results did not support this standpoint.

## 6. Experiment 5

In this experiment, we further explored the role of conceptual distinctiveness of real-world objects on the VWM task performance by setting two different encoding times identical to Experiment 2 (100 vs. 1000 ms). We expected that the null effect of conceptual distinctiveness would be replicated in this version.

### 6.1. Methods

#### 6.1.1. Participants

Twenty-five participants (five males; mean age: 21.76 years) took part in the current experiment. The requirements for subjects were identical to Experiment 2.

#### 6.1.2. Apparatuses and stimuli

Apparatuses and stimuli were identical to Experiment 4.

#### 6.1.3. Procedure

The current procedure was identical to Experiment 2. However, we set 3 blocks of 144 trials each in the formal testing phase.

### 6.2. Results and discussion

**FIGURE 11:**
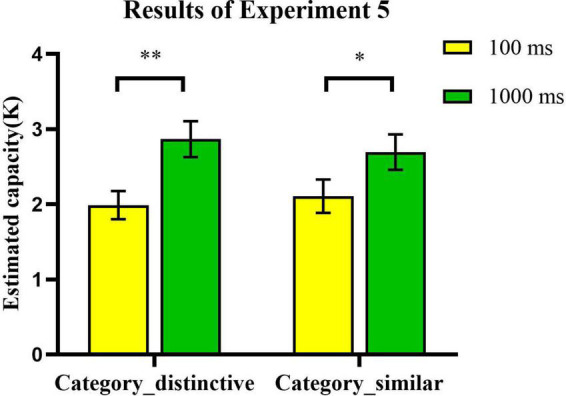
The effect of conceptual distinctiveness and encoding time on the VWM task performance. Error bars reflect standard error of mean. *, 0.01 < *p* < 0.05; **, 0.001 < *p* < 0.01; ***, *p* ≤ 0.001; and ****, *p* ≤ 0.0001.

We constructed a 2 × 2 (encoding time × type of real-world objects) repeated measures analysis of variance (ANOVA) for the current experiment. The results showed that there were significant main effects for encoding time [*F*_(1_, _24)_ = 17.55, *p* < 0.001, η^2^_*p*_ = 0.42, BF_10_ = 71.663]. Subsequent analysis showed that the capacities were higher in the 1000 ms (*Mean*: *K* = 2.78, *SEM* = 21%) than in the 100 ms condition (*Mean*: *K* = 2.05, *SEM* = 16%). In addition, we replicated the results of Experiment 4. We found no significant main effect for conceptual information [*F*_(1_, _24)_ = 0.02, *p* = 0.882, η^2^_*p*_ = 0.001, BF_10_ = 0.276]. There were no interaction effects between encoding time and type of real-world objects [*F*_(1_, _24)_ = 1.07, *p* = 0.311, η^2^_*p*_ = 0.043, BF_10_ = 0.484] (Figure 11). We replicated the findings of Experiment 4, showing that the conceptual distinctiveness of real-world objects did not affect VWM storage regardless of the length of encoding time.

## 7. General discussion

In the experiments presented here, we started to explore the role of perceptual information in real-world objects storage in VWM. As shown in Experiments 1 through 3, the color and shape distinctiveness of real-world objects impacted VWM capacity. VWM capacity was enhanced by real-world objects which were perceptually distinctive on change detection tasks. However, in Experiments 4 to 5, we did not find an effect of conceptual distinctiveness on VWM capacity. Although we did not find a significant encoding time benefit in Experiment 2, the effect of color distinctiveness remained significant. In summary, these results indicated that VWM capacity could benefit from the features of real-world objects, which were perceptually distinctive without reference to encoding time. VWM capacity might not be affected by real-world objects’ conceptual distinctiveness.

Research has focused on the effects of objects’ perceptual information on VWM capacity. Alvarez and Cavanagh (2004) explored the VWM capacity of six different classes of objects. Their results demonstrated that VWM capacity was not fixed. It varied across classes of stimulus materials with differing information loads. Similarly, Eng et al. (2005) found that VWM capacity declined significantly for objects having more complex perceptual information. These studies found a correlation between visual search rate and information load per item (e.g., Konkle et al., 2010), and their results suggested that perceptual complexity impacts VWM processes. In the current Experiments 1 to 3, we selected real-world objects containing different color and shape properties as experiment stimuli. These manipulations could result in less information load on memory from objects which were perceptually distinctive. These different information loads are reflected in the current results in different conditions (e.g., *color_distinctive* vs. *color_similar* conditions). Although the insufficient encoding time might lead to a limited encoding process for perceptually similar objects, we excluded this possibility in Experiments 2 and 3 by prolonging the stimulus display times.

There was also another possible explanation for the results of Experiments 1, 2, and 3. We hypothesized that, as a result of different perceptual properties of memory stimuli, the memory fidelity requirements would vary. Specifically, low-fidelity representations could meet change detection tasks requirements in conditions that featured perceptually distinctive memory items. However, participants had to maintain high-fidelity representations of objects when required to memorize perceptually similar items because participants could not rely on feature information to make correct judgments in change detection tasks. For instance, people could rely on the color information of objects to make correct judgments when memory items were gifts (*color-distinctive* condition). However, when memory items were jack o’lanterns (*color-similar* condition), people could not simply rely on the color information about objects. They required detailed information about the objects. High-fidelity representations would occupy more cognitive resources. Thus, they resulted in a reduction in the number of memory items in perceptual similarity conditions (Wilken and Ma, 2004; Bays and Husain, 2008; Zhang and Luck, 2008; Bays et al., 2009).

There has been much research on categories as the organizing structure for long-term verbal memory (Collins and Loftus, 1975; Mervis and Rosch, 1981; Jolicoeur et al., 1984). Several studies have shown that concepts connecting to existing knowledge about objects such as human faces or semantic information could support visual memory (Wiseman and Neisser, 1974; Koutstaal et al., 2003). Konkle et al. (2010) further demonstrated that the capacity for real-world objects in long-term memory depended on conceptual structures. Brady et al. (2011) suggested that categorical dimensions of real-world objects might provide “conceptual hooks” to recover memory traces in LTM and increase the probability of successful retrieval. There were similar conclusions in the field of VWM. For example, Olsson and Poom (2005) supposed that objects and colors typically used in VWM studies were easily classified into distinct categories and helped participants develop category boundaries. This categorical information contaminated the estimated VWM capacity.

In contrast, in Experiments 4 to 5, we explored the effects of conceptual distinctiveness of real-world objects on VWM capacity. The results showed that the conceptual distinctiveness of objects did not significantly impact VWM capacity. Based on these contradictory conclusions, we supposed that such incompatible results were related to differences in VWM storage mechanism compared to LTM (e.g., Norris, 2017). However, there have been many behavioral and neuroimaging studies providing evidence for shared principles of short- and long-term memory (e.g., Jonides et al., 2008). Berryhill and Olson (2008) demonstrated that the posterior parietal cortex (PPC) plays a particularly vital role in working memory retrieval. PPC activity has been proposed as a link to LTM processes (e.g., Vilberg and Rugg, 2008). Similarly, there has been some research supporting the hypothesis that short-term memory is nothing more than activated LTM (e.g., Cowan, 1988, 1999; Oberauer, 2002). Our results did not support these ideas. On the one hand, we supposed that real-world objects might have multi-dimensional categorical information. This complexity of categorical information might make retrieval difficult in VWM tasks. Real-world objects possess not only perceptual features such as color and shape but also functional and social information (e.g., Kaiser et al., 2015). Inconsistent with LTM, this complex categorical information might cause short-duration VWM retrieval confusion. On the other hand, we adopted six exemplars randomly selected from one object category in each trial to prevent cross-category effects in VWM storage (Awh et al., 2007). This manipulation might have weakened the role of conceptual information in retrieval because all objects in the sample display phase were belonging to one exact given objects. Although we prolonged the encoding time in the sample display phase, the null-effect of conceptual distinctiveness on VWM was still confirmed. Similarly, Markov and Utochkin (2021) used low- and high-conceptual distinctiveness real-world object samples from Konkle et al. (2010) to require participants to finish a working memory recognition task. The results showed that there were no effects of object distinctiveness on correct memory rate or *P*_*guess*_. The operational definition for conceptual distinctiveness of objects adopted here differs from Markov and Utochkin (2021), yet our findings were consistent with theirs.

Based on all findings, we surmise that VWM processes for real-world objects might be dependent on what information is encoded in the visual system and the passive information stored in LTM might not be involved in these processes automatically. Kaiser et al. (2015) first investigated the spatial positional relationship of real-world objects on VWM performance and they found that the VWM performance benefit would be gained from the object which conforms to the spatial regularity in a real-life setting (e.g., a lamp above the table) compared to the object without spatial regularity (e.g., a lamp below the table). Furthermore, Liu et al. (2022) conducted an examination of the specific VWM phase of spatial regularities effect for real-world objects and they found that the effect of spatial regularity occurred only when objects presented simultaneously but not presented sequentially. Liu et al. (2022) concluded that the benefit of spatial regularity for real-world objects in VWM conforms to the “encoding specificity” hypothesis, which suggested that VWM representations were determined by how information were encoded when visual stimuli appear (Tulving and Thomson, 1973; Woodman et al., 2003). Liu et al. (2022) research provided evidence that the VWM process might depend on more current information engaged other than information passively stored in VLTM. Thus, according to the current research, perceptual information for real-world objects could be rapidly processed into representations following the bottom-up principle. However, conceptual information about real-world objects is not extracted in the same way. Our results provided evidence that VWM processes for real-world objects might follow a bottom-up principle. Existing knowledge stored in the LTM system may not be involved in this process.

Our study has some limitations. In current study, we directly distinguished the perceptual and conceptual distinctiveness of real-world objects by scale rating quoted from Konkle et al. (2010). However, this manipulation might not be sufficient to exclude the irrelevant variables that might affect the outcomes. For example, in Experiment 1, there were two pairs of matched categories selected from the pool: buttons and camcorders, gifts and jack o’lanterns, although we controlled the matched dimensions (i.e., kind and shape information) for these two paired objects as much as possible, there is no denying that the kind or shape diversity of real-world objects could still cause some level of confounding in the outcomes. It must be acknowledged that the diversity of perceptual and conceptual information is an inherent attribute of any given real-world object. Future studies could pay more attention to the diversity of operational definitions for the distinctiveness of conceptual and perceptual information of real-world objects.

## Data availability statement

The raw data supporting the conclusions of this article will be made available by the authors, without undue reservation.

## Ethics statement

The studies involving humans were approved by the Scientific and Ethical Review Committee of the Department of Psychology of Zhejiang Normal University. The studies were conducted in accordance with the local legislation and institutional requirements. The participants provided their written informed consent to participate in this study.

## Author contributions

All authors listed have made a substantial, direct, and intellectual contribution to the work, and approved it for publication.

## Footnotes

1
https://bradylab.ucsd.edu/stimuli.html
